# Machine Learning-Based Automatic Diagnosis of Osteoporosis Using Bone Mineral Density Measurements

**DOI:** 10.3390/jcm15020549

**Published:** 2026-01-09

**Authors:** Nilüfer Aygün Bilecik, Levent Uğur, Erol Öten, Mustafa Çapraz

**Affiliations:** 1 Department of Physical Therapy and Rehabilitation, Adana City Training and Research Hospital, Adana 01370, Turkey; 2 Department of Mechanical Engineering, Faculty of Engineering, Amasya University, Amasya 05100, Turkey; 3 Department of Physical Therapy and Rehabilitation, Faculty of Medicine, Amasya University, Amasya 05100, Turkey; 4 Department of Internal Medicine, Faculty of Medicine, Amasya University, Amasya 05100, Turkey

**Keywords:** classification, machine learning, BMD, DXA

## Abstract

**Background:** Osteoporosis and osteopenia are prevalent bone diseases characterized by reduced bone mineral density (BMD) and an increased risk of fractures, particularly in postmenopausal women. While dual-energy X-ray absorptiometry (DXA) remains the gold standard for diagnosis, it has limitations regarding accessibility, cost, and predictive capacity for fracture risk. Machine learning (ML) approaches offer an opportunity to develop automated and more accurate diagnostic models by incorporating both BMD values and clinical variables. **Method:** This study retrospectively analyzed BMD data from 142 postmenopausal women, classified into 3 diagnostic groups: normal, osteopenia, and osteoporosis. Various supervised ML algorithms—including Support Vector Machines (SVM), k-Nearest Neighbors (k-NN), Decision Trees (DT), Naive Bayes (NB), Linear Discriminant Analysis (LDA), and Artificial Neural Networks (ANN)—were applied. Feature selection techniques such as ANOVA, CHI2, MRMR, and Kruskal–Wallis were used to enhance model performance, reduce dimensionality, and improve interpretability. Model performance was evaluated using 10-fold cross-validation based on accuracy, true positive rate (TPR), false negative rate (FNR), and AUC values. **Results:** Among all models and feature selection combinations, SVM with ANOVA-selected features achieved the highest classification accuracy (94.30%) and 100% TPR for the normal class. Feature sets based on traditional diagnostic regions (L1–L4, femoral neck, total femur) also showed high accuracy (up to 90.70%) but were generally outperformed by statistically selected features. CHI2 and MRMR methods also yielded robust results, particularly when paired with SVM and k-NN classifiers. The results highlight the effectiveness of combining statistical feature selection with ML to enhance diagnostic precision for osteoporosis and osteopenia. **Conclusions:** Machine learning algorithms, when integrated with data-driven feature selection strategies, provide a promising framework for automated classification of osteoporosis and osteopenia based on BMD data. ANOVA emerged as the most effective feature selection method, yielding superior accuracy across all classifiers. These findings support the integration of ML-based decision support tools into clinical workflows to facilitate early diagnosis and personalized treatment planning. Future studies should explore more diverse and larger datasets, incorporating genetic, lifestyle, and hormonal factors for further model enhancement.

## 1. Introduction

Osteoporosis and osteopenia are common bone diseases affecting millions of people worldwide, characterized by low bone mineral density (BMD) and microstructural deterioration of bone tissue [1]. It is among the most common diseases in elderly populations [2,3], with the global population projected to increase by more than 50% to more than 2 billion by 2050 (6). The World Health Organization (WHO) defines osteoporosis as 2.5 standard deviations below the mean young adult BMD [4,5]. However, because osteoporosis usually follows an asymptomatic course, most patients are diagnosed only after a fracture has developed [6]. Fractures resulting from osteoporosis constitute a significant public health problem, causing serious morbidity and mortality, especially in elderly individuals [7]. Therefore, early diagnosis of the disease and identification of high-risk individuals are critical.

BMD measurement is one of the most widely used methods for the diagnosis of osteoporosis, usually performed with dual-energy X-ray absorptiometry (DXA) scans [8]. Although DXA is considered the gold standard for the diagnosis of osteoporosis, it has some important limitations. The high cost of these scans, limited access, and the fact that they are applicable only to certain patient groups make it difficult to spread screening programs in large segments of the population [9]. In addition, DXA provides an assessment based only on BMD values, which may cause it to be inadequate in determining the risk of osteoporosis. Studies have shown that DXA alone may not be sufficient to estimate the true fracture risk of individuals, and additional variables such as age, gender, genetic factors, and lifestyle should also be evaluated [10].

These difficulties reveal the need to determine a personalized osteoporosis diagnosis and treatment strategy, taking into account patient characteristics and individual risk factors. Currently, in clinical practice, there is no definitive approach that can predict which treatment will respond better to individuals. This creates uncertainty about which treatment protocol should be applied, especially in individuals diagnosed early [11]. Therefore, there is a need to develop new and more advanced methods that can evaluate the risk of osteoporosis based on individual characteristics of patients and guide the selection of optimal treatment.

Machine learning (ML) and artificial intelligence (AI) based methods are increasingly used in medical diagnosis processes and provide higher accuracy rates compared to traditional methods, especially by analyzing large data sets [11]. In osteoporosis diagnosis, supervised learning methods such as support vector machines (SVM), random forests (RF) and artificial neural networks (ANN) offer promising results, especially in classification processes, reaching high accuracy rates [12]. Deep learning based models are also coming into play to overcome the limitations of traditional BMD measurements and offer a new approach to determine osteoporosis risk by estimating BMD from computed tomography (CT) and magnetic resonance imaging (MRI) data [7].

In line with these developments, AI-supported models have the potential to provide more accurate and personalized diagnosis and treatment recommendations not only based on BMD measurements, but also by taking into account patient history, clinical data, biomarkers and genetic factors [13]. In particular, by performing data analysis specific to the patient profile, it offers the opportunity to improve early diagnosis processes for individuals at high risk of osteoporosis and to create personalized treatment approaches [14]. In this way, it can provide doctors with stronger and data-supported decision-making mechanisms in the process of determining the most appropriate treatment option for the individual’s bone health.

This study aims to implement and evaluate machine learning techniques for the classification of osteoporosis, osteopenia, and healthy individuals using BMD data. In particular, the goal is to identify the most accurate model by comparing the performance of multiple classification algorithms integrated with different feature selection methods. While previous studies have applied ML models to similar problems, many rely on complex deep learning systems or CT/MRI data that are not widely available. In contrast, this study uses only standard DXA-derived BMD measurements, offering a low-cost, accessible, and interpretable approach. A key contribution lies in the systematic comparison of statistical feature selection techniques (ANOVA, CHI2, MRMR, Kruskal–Wallis) against clinically preferred anatomical regions. This allows us to demonstrate that data-driven features can outperform conventional diagnostic sites in classification accuracy. The proposed method not only simplifies the diagnostic process but also offers a reproducible and automated framework that can be readily integrated into routine clinical workflows.

## 2. Materials and Methods

### 2.1. Patient Characteristics

This study includes data from patients who applied to the Physical Therapy and Rehabilitation Polyclinic of Amasya University Faculty of Medicine between December 2021 and July 2025 and underwent bone mineral density (BMD) evaluation. Patients with diabetes, gout, rheumatoid arthritis, or other systemic diseases that could affect osteoporosis diagnosis were excluded. In addition, individuals with a history of trauma or surgery likely to alter bone structure were not considered. A total of 1500 BMD measurements were retrospectively reviewed, and data from 142 patients met the inclusion criteria and were analyzed.

All 142 patients included in this study were postmenopausal women (mean age 60.4 ± 9.1 years), a group particularly susceptible to accelerated bone loss. Each individual was classified as normal, osteopenic, or osteoporotic based on bone mineral density (BMD) measurements obtained from DXA (Dual-energy X-ray Absorptiometry) scans. The classification was performed according to the diagnostic thresholds defined by the World Health Organization (WHO): T-scores ≥ −1.0 were considered normal, between −1.0 and −2.5 as osteopenia, and ≤−2.5 as osteoporosis [15]. Based on these criteria, 40 patients were categorized as normal, 52 as osteopenic, and 50 as osteoporotic. Ethics committee approval for this study was obtained from Amasya University Ethics Committee (2025/18). The demographic and clinical characteristics of the study population are presented in Table 1.

DXA measurements were performed using a standard DXA device (GE Lunar Prodigy, GE Healthcare, Madison, WI, USA) with a standardized protocol during routine clinical examinations at Amasya University Faculty of Medicine. The DXA images were automatically analyzed, and reports were generated using vendor-specific software (Lunar DPX–enCORE 2010, version 13.31.015; General Electric (GE), Madison, WI, USA).

**Figure 1:**
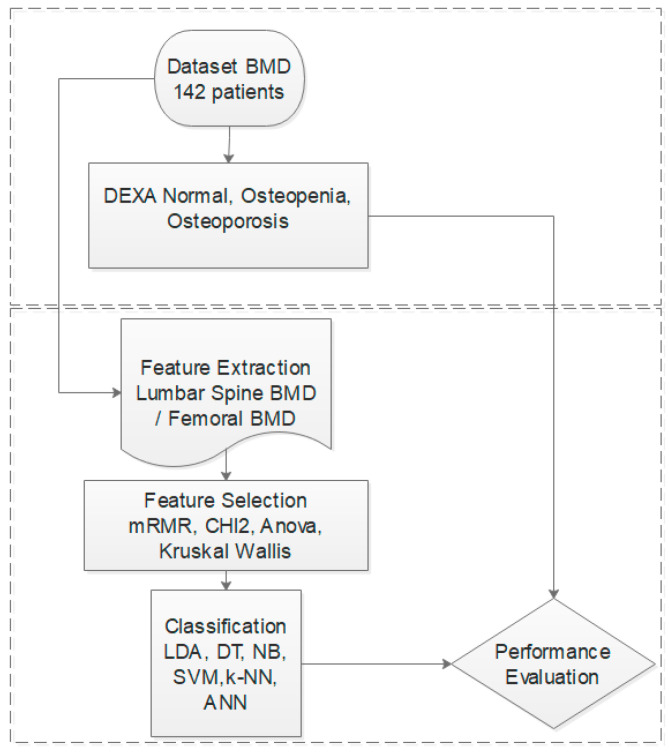
Flow chart of this study.

The diagram presented in Figure 1 illustrates the methodological workflow followed in this study for classifying BMD data using machine learning techniques. This systematic approach, as shown in the diagram, clearly outlines the process from data acquisition to performance evaluation, ensuring that each step contributes to enhancing diagnostic accuracy and clinical applicability in osteoporosis management.

### 2.2. BMD Classification and Evaluation Process

The diagnosis of osteoporosis is commonly based on BMD measurements, with dual-energy X-ray absorptiometry DXA being the most frequently used technique for this purpose. DXA assessments are typically performed using BMD values obtained from the lumbar spine (L1–L4), femoral neck, trochanter, and total femur regions. According to the WHO criteria, individuals with a BMD T-score of −1.0 or above are classified as normal, those with a T-score between −1.0 and −2.5 as osteopenic, and those with a T-score of −2.5 or below as osteoporotic [16].

In clinical practice, the most commonly used parameters for diagnosis include the L1–L4 mean, femoral neck, and total femur values. However, since lumbar spine measurements may be influenced by age-related degenerative changes, femoral region measurements (particularly the neck and total femur) are considered to provide more stable and reliable results [17]. To enhance diagnostic accuracy, several studies have emphasized the importance of using the mean T-score of the L1–L4 region rather than relying on individual vertebrae [18]. Additionally, it has been reported that T-scores may differ between the lumbar spine and hip regions, potentially leading to diagnostic inconsistencies [19].

In this study, classification was performed using BMD values obtained from the L1, L2, L3, L4, L1–L2, L1–L3, L1–L4, L2–L3, L2–L4, L3–L4, and femoral regions (neck, trochanter, and total femur). The literature indicates that BMD measurements from different anatomical sites can have varying impacts on the diagnosis of osteoporosis [20]. For instance, lumbar vertebral measurements may be affected by age-related degenerative changes, while femoral neck and total femur BMD values are more directly associated with osteoporosis risk [21].

Nevertheless, the accuracy and reliability of the DXA results may vary depending on the device used and the anatomical region measured. For example, different DXA systems, such as Lunar iDXA and Horizon A, may yield differing BMD and T-score results for the same individual. These differences can directly affect the classification of individuals as osteoporotic or osteopenic. One study reported that although the Lunar iDXA device recorded higher BMD values compared to the Horizon A system, it yielded lower T-scores. Despite an overall 82% agreement between these two devices in osteoporosis diagnosis, individual-level discrepancies in classification were observed.

In the present study, the dataset was divided into three subsets: 70% of the data was used for training, 15% for validation, and 15% for testing. This data partitioning strategy was selected to ensure adequate learning during the training phase while also allowing for robust validation and testing processes. Furthermore, k-fold cross-validation was employed to enhance the performance and generalizability of the model [20].

### 2.3. Feature Extraction and Dimensionality Reduction

In this study, a comprehensive feature extraction and dimensionality reduction process was applied to BMD data obtained via DXA devices to enhance the accuracy of classification models and strengthen the model’s generalization capability. In addition to BMD measurements from the lumbar spine (L1–L4), femoral neck, trochanter, and total femur regions, demographic and anthropometric variables such as age, height, weight, and BMI were also included as features.

Using T-scores derived from each anatomical site, the mean, minimum, and maximum BMD values were calculated. Based on the WHO criteria, individuals were categorized into diagnostic groups (normal, osteopenic, or osteoporotic), and these classifications were modeled as labels.

During the data preprocessing phase, highly correlated features were identified and removed to avoid overfitting. All numerical variables were scaled using Z-score normalization. Feature selection and dimensionality reduction were implemented to reduce model complexity, enhance interpretability, and improve computational efficiency by eliminating redundant or irrelevant data. In this context, various statistical and algorithmic methods such as Minimum Redundancy Maximum Relevance (MRMR), Chi-square test (Chi2), ANOVA F-test, and Kruskal–Wallis test were employed. Principal Component Analysis (PCA) was also used to reduce the high-dimensional data structure into a smaller set of components, thereby improving classifier performance. As reported in the literature, such techniques are highly effective in increasing model accuracy and reducing the risk of overfitting [22,23]

### 2.4. Classification

The classification task in this study aimed to accurately categorize individuals as normal, osteopenic, or osteoporotic based on their bone mineral density (BMD) data. Within the context of feature selection, classification involves identifying the most meaningful and discriminative features to improve the overall performance of the model. For this purpose, supervised learning algorithms were utilized, and analyses were conducted separately on both the original high-dimensional dataset and the reduced-dimensional dataset.

By eliminating noisy and irrelevant data, the computational burden of the model was reduced, while classification accuracy was improved [24,25]. The classification algorithms employed in this study included Decision Tree (DT), Linear Discriminant Analysis (LDA), Naive Bayes (NB), k-Nearest Neighbors (k-NN), Support Vector Machines (SVM), and Artificial Neural Networks (ANN). These algorithms were evaluated under various feature selection and dimensionality reduction scenarios, and their performances were compared accordingly.

To assess the accuracy and generalizability of the models, a 10-fold cross-validation approach was adopted. The success rates of each classifier were analyzed in both the original and reduced feature spaces. This multi-faceted approach enabled the identification of the most effective classification model and supported the development of a reliable artificial intelligence-based system for early osteoporosis diagnosis. While 10-fold cross-validation was used to reduce overfitting, no external validation set was available in this study. Future research should include independent datasets to better assess generalizability across populations and clinical settings.

All classifiers were implemented using MATLAB’s Classification Learner App. For each algorithm (Version R2023b, MathWorks, Natick, MA, USA), hyperparameter tuning was performed using Bayesian optimization when applicable. For instance, the SVM classifier utilized a radial basis function (RBF) kernel, with box constraint and kernel scale parameters automatically optimized. The k-NN model was optimized for the number of neighbors and distance metric. In decision trees, the maximum tree depth and minimum leaf size were adjusted. The artificial neural network (ANN) model was a feedforward network trained using scaled conjugate gradient backpropagation with a single hidden layer. Naive Bayes assumed Gaussian distribution for each class. These settings ensured optimal model configuration under the 10-fold cross-validation framework.

### 2.5. Statistical Analysis

All statistical analyses were conducted using IBM SPSS Statistics version 25.0. The Shapiro–Wilk test was employed to assess the normality of data distribution. Continuous variables with a normal distribution are presented as mean ± standard deviation, whereas those not normally distributed are presented as median (interquartile range). For group comparisons, one-way ANOVA was used for normally distributed data, while the Kruskal–Wallis test was applied for non-normally distributed data. When the ANOVA results were significant, post hoc comparisons were performed using Tukey’s HSD test. The chi-square test was used to compare categorical variables.

For the evaluation of machine learning model performance, accuracy, sensitivity, specificity, and the area under the receiver operating characteristic curve (AUC) were calculated. A *p*-value of less than 0.05 was considered statistically significant in all analyses.

## 3. Results

This section presents the performance outcomes of the classification models developed based on BMD data obtained through DXA. The analyses incorporated measurements from different anatomical sites (L1–L4, femoral neck, trochanter, and total femur), along with additional clinical parameters such as age and BMI. The effects of applied feature selection and dimensionality reduction techniques on classification accuracy were examined in detail.

Each classifier algorithm (Decision Tree [DT], Linear Discriminant Analysis [LDA], Naive Bayes [NB], k-Nearest Neighbors [k-NN], Support Vector Machines [SVM], and Artificial Neural Networks [ANN]) was tested using a 10-fold cross-validation method on both the original high-dimensional dataset and the dimensionally reduced datasets. The obtained results highlight which algorithm and preprocessing strategy were more effective in classifying osteoporosis, osteopenia, and normal bone conditions, thereby supporting the clinical potential of the proposed system for early diagnosis.

The performance results of the classifiers using all features are presented in Table 2. In terms of overall accuracy, the highest performance was observed with the DT and NB algorithms, both achieving 82.90%. These were followed by SVM (75.00%), ANN (74.30%), and k-NN (72.90%), while the lowest accuracy was obtained with LDA at 70.00%.

For the classification of actual normal cases. SVM achieved the highest true positive rate (TPR) at 95.20%. In the osteopenia class, LDA was the most successful model with a TPR of 90.00%, whereas for the osteoporosis class, both k-NN and ANN achieved the highest TPR at 87.50%.

**Figure 2:**
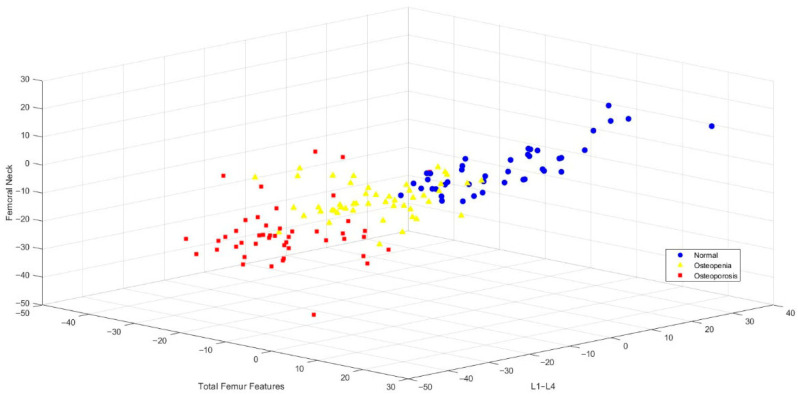
Visualized Distribution of BMD Groups in 3D Space According to L1–L4, Femoral Neck and Total Femur Features.

In this study, classification analysis was performed based on three primary BMD measurement sites—L1–L4, femoral neck, and total femur—which are commonly used by clinicians for diagnostic decision-making. The spatial distribution of these features was visualized (Figure 2). The 3D scatter plot revealed distinct clustering patterns among individuals categorized as normal (blue), osteopenic (yellow), and osteoporotic (red), supporting the discriminative power of these three anatomical regions in osteoporosis classification.

The classification models developed using the selected features are summarized in Table 3. According to the results, the SVM, k-NN, and NB algorithms demonstrated the highest overall accuracy rates, with 90.70%, 90.00%, and 89.30%, respectively. All models achieved high true positive rates (TPRs) for the osteoporosis class—e.g., 95.80% in LDA, NB, and SVM—indicating strong classification performance for this diagnostic category.

However. model performance varied across algorithms for the normal and osteopenia classes. In particular, the k-NN algorithm achieved outstanding performance for the normal class, with a TPR of 97.60%, while the NB model was more successful for the osteopenia class, reaching a TPR of 78.00%.

These findings suggest that the three anatomical regions—L1–L4, femoral neck, and total femur—play a critical role in diagnosis and can be effectively integrated into machine learning models to build robust classifiers, thereby providing valuable decision support for clinicians in osteoporosis diagnosis.

**Figure 3:**
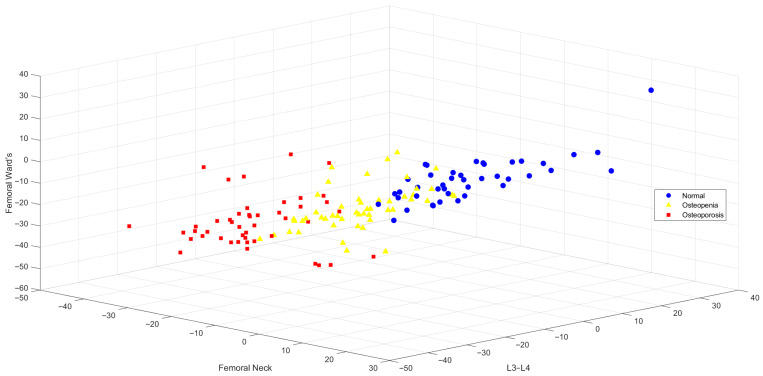
3D Visualization of BMD Group Distributions Based on L3–L4, Femoral Neck, and Femoral Ward’s Features.

The classification results obtained using the most relevant and least redundant variables selected by the Minimum Redundancy Maximum Relevance (MRMR) feature selection method are presented in Table 4. In this method, BMD values from the L3–L4, femoral neck, and femoral Ward’s regions were identified as the most discriminative features. The three-dimensional distribution of these variables is visualized in Figure 3. Upon examination of Figure 3, it is evident that individuals classified as normal, osteopenic, and osteoporotic are clustered into distinct groups, indicating that the selected features effectively support inter-group differentiation.

Regarding the performance of classification algorithms using MRMR-selected features, the highest overall accuracy was achieved by the DT and SVM models, both reaching 82.90%. These were followed by Naive Bayes (NB) at 80.00%, LDA at 78.60%, k-NN at 77.90%, and ANN at 75.70%. While all models demonstrated high TPRs for the osteoporosis class (ranging from 77.10% to 89.60%), the performance for the osteopenia class remained relatively lower across algorithms.

**Figure 4:**
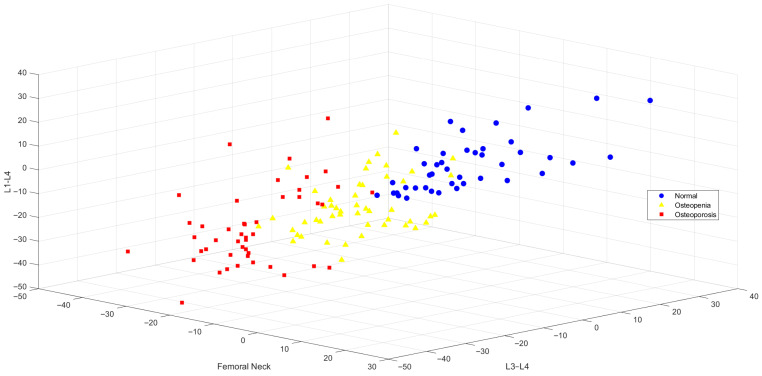
3D Visualization of BMD Group Distributions Based on L3–L4, Femoral Neck, and L1–L4 Features.

Following feature selection using the Chi-Square (CHI2) method, the variables with the highest information gain were identified as L1–L4, L3–L4, and femoral neck BMD values. The distribution of these selected features in three-dimensional space is visualized in Figure 4. The figure reveals that individuals in the normal, osteopenic, and osteoporotic categories are positioned in clearly distinguishable clusters, highlighting the discriminative contribution of CHI2-selected variables in class separation.

The classification performance results based on CHI2 feature selection are presented in Table 5. The highest overall accuracy was achieved by the SVM algorithm at 91.40%, followed by k-NN at 90.70%, and both DT and ANN at 90.00%. Notably, SVM and k-NN achieved 100% TPR for the normal class.

**Figure 5:**
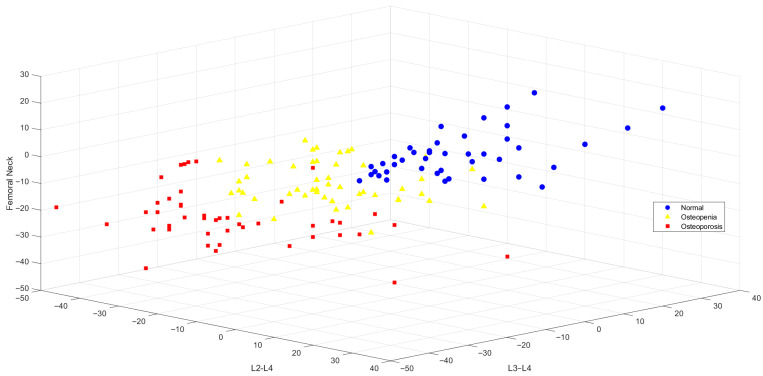
Distribution of Selected Features (L2–L4, L3–L4, Femoral Neck) in 3D Space with ANOVA.

Following feature selection using the ANOVA method, the variables that best represented the inter-group variance were identified as L2–L4, L3–L4, and femoral neck BMD values. The three-dimensional distribution of these features is illustrated in Figure 5. The visualization demonstrates a clear separation among the classes, indicating that the selected variables provide a strong foundation for diagnostic discrimination.

The classification performance results of models developed using these features are detailed in Table 6. The SVM algorithm achieved the highest performance, with 94.30% accuracy and a 100% true positive rate (TPR) for the normal class.

**Figure 6:**
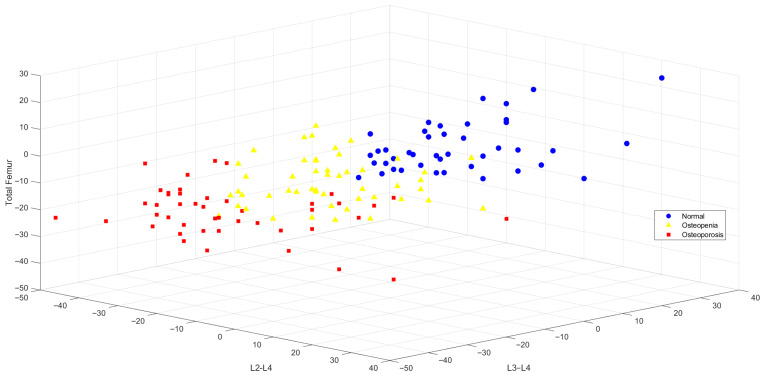
3D Distribution of Features Selected via Kruskal–Wallis Test (L2–L4, L3–L4, Total Femur).

Following feature selection using the Kruskal–Wallis test, the most prominent variables were identified as L2–L4, L3–L4, and total femur BMD values. The three-dimensional distribution of these features is visualized in Figure 6. The plot reveals distinct clustering of normal, osteopenic, and osteoporotic individuals in the feature space, with the osteoporotic group notably concentrated in the lower regions.

According to the classification results presented in Table 7, the highest overall accuracy was achieved by the SVM and k-NN algorithms, both reaching 85.00%. These two models also demonstrated strong class separation for the normal group, with TPRs of 92.90% for SVM and 95.20% for k-NN, respectively.

A comparative analysis of the overall accuracy rates obtained from all classifiers and various feature selection methods is presented in Table 8. The highest classification accuracy (94.30%) was achieved by the SVM algorithm when ANOVA-based feature selection was applied. This was followed by k-NN (92.10%) and ANN (91.40%), also using ANOVA-selected features. Overall, ANOVA-based feature selection provided the most consistent and highest accuracy results across all classifiers.

Interestingly, high accuracy rates were also observed when using features derived from anatomical regions commonly employed in clinical practice—namely, L1–L4, femoral neck, and total femur—which are typically referred to as the “standard diagnostic regions.” For example, classification using these features yielded accuracy rates of 90.70% (SVM), 90.00% (k-NN), and 89.30% (Naive Bayes). However, these results were generally slightly lower than those obtained through statistically driven feature selection methods, particularly ANOVA.

This finding suggests that while clinically preferred regions offer a strong foundation for classification, statistically selected features—such as those identified by ANOVA—may provide superior discriminative power. Additionally, the Chi-Square (CHI2) method also yielded competitive performance, achieving 91.40% accuracy with SVM, which was comparable to ANOVA.

This comparative analysis clearly demonstrates that feature selection strategy is a critical factor in classification performance, highlighting the potential of data-driven approaches to surpass traditional clinical assumptions.

To further assess the performance of the best-performing model (SVM with ANOVA-selected features), detailed classification metrics including precision, recall, and F1-score were calculated for each class. As shown in Table 9, the model achieved exceptionally high recall (1.000) for the Normal class, along with strong precision and F1-scores across all categories (e.g., 0.978 precision for Osteoporosis). The macro-averaged precision, recall, and F1-score were all above 0.94, indicating balanced classification ability despite the multiclass structure and moderate class imbalance in the dataset.

**Figure 7:**
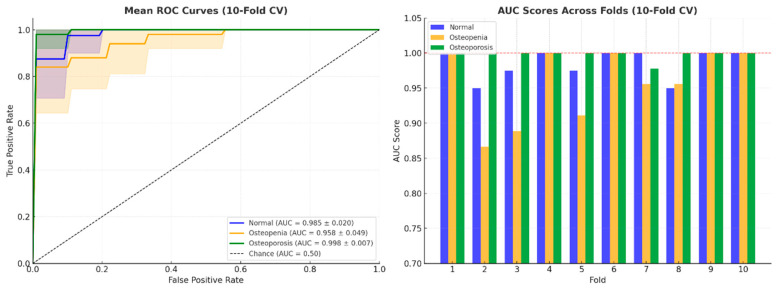
ROC curves (**left**) and AUC scores across 10-fold cross-validation (**right**) for the SVM classifier using ANOVA-selected features.

In addition, receiver operating characteristic (ROC) curves and area under the curve (AUC) values were computed through 10-fold cross-validation to evaluate class-wise discrimination. As illustrated in Figure 7, the model demonstrated excellent separability, achieving AUC scores of 0.984 for Normal, 0.964 for Osteopenia, and 0.996 for Osteoporosis. The AUC bar chart further confirms the model’s robustness, showing consistently high scores across all folds for each class. These findings collectively reinforce the SVM + ANOVA model’s suitability as a reliable and generalizable classifier for early osteoporosis detection.

## 4. Discussion

In this study, the effectiveness of machine learning algorithms in the classification of bone mineral density (BMD) data for the diagnosis of osteoporosis and osteopenia was investigated, and the impact of various feature selection methods was compared. According to the findings, variables selected through the ANOVA (Analysis of Variance) method achieved the highest overall accuracy rates across all classifiers, with the SVM algorithm reaching a peak performance of 94.30% accuracy.

This result is consistent with recent studies in the literature. For instance, in a study conducted by Miranda et al. (2022), ANOVA-based feature selection, when combined with clinical and ultrasound data, significantly improved the classification success of patients, surpassing the predictive power of traditional DXA-based features [26].

In addition, Khanna et al. (2023) emphasized that statistical selection techniques such as ANOVA enhance not only the accuracy but also the interpretability of classifiers when integrated with explainable artificial intelligence (XAI) tools like SHAP and LIME [27]. Similarly, the CLIF framework proposed by Calitis (2024) further validated the role of ANOVA in identifying clinically meaningful feature groups, showing that the integration of feature selection with ablation testing significantly boosts classification performance. [28] These findings collectively underline the importance of statistically grounded, data-driven feature selection approaches as a foundation for developing accurate, interpretable, and clinically reliable AI-based decision support systems in osteoporosis diagnosis.

This study also demonstrated that the conventional diagnostic approach based on clinically preferred regions—L1–L4, femoral neck, and total femur—offers relatively high classification accuracy, with the SVM algorithm achieving 90.70% accuracy using these features. However, features selected through statistical methods generally resulted in more balanced classification outcomes across diagnostic classes. This finding is supported by a study based on data from the Korean National Health and Nutrition Examination Survey, which reported that variables such as age, BMI, and alcohol consumption exhibited varying levels of importance across genders. The authors noted that machine learning-based feature selection was better equipped to model such heterogeneous effects [29] (JBM, 2023).

Beyond integration into clinical workflows, it is also essential to compare the model’s diagnostic capability with established fracture risk assessment tools such as FRAX. Although FRAX remains a widely accepted method, several studies have pointed out its limitations. For example, Oka et al. (2017) found that FRAX without BMD lacked sensitivity in middle-aged populations [30], while Sheng et al. (2024) reported only moderate alignment between FRAX scores and actual fracture outcomes over an 11-year follow-up [31]. Recent evidence by Lehmann et al. (2024) demonstrated that ML models could outperform FRAX in fracture prediction, especially when enhanced with explainable AI tools such as SHAP [32]. These comparisons highlight the need for dynamic, data-driven diagnostic alternatives.

From a clinical standpoint, such models offer promising decision-support capabilities. The algorithm could be integrated into existing diagnostic workflows to assist radiologists and endocrinologists in cases where T-scores are borderline or ambiguous. It could serve as an auxiliary screening layer, automatically highlighting at-risk individuals based on multiple BMD inputs, reducing manual variability, and potentially enhancing early intervention strategies. Moreover, the model’s ability to generalize across reduced feature spaces suggests feasibility for implementation in settings where only limited anatomical regions are available.

Despite these promising results, several limitations should be acknowledged. The dataset was derived from a single center (Amasya University) and lacked participants from diverse geographic or ethnic backgrounds, which may limit the generalizability of the findings. Device-related variability is another important limitation, as different DXA systems may produce varying BMD and T-score values for the same individual, potentially affecting the performance and reliability of classification models across clinical settings. Moreover, the analysis relied exclusively on BMD data and did not include other osteoporosis risk factors such as lifestyle habits, hormonal status, or genetic predisposition. Although dimensionality reduction techniques were applied to improve model efficiency, some methods showed limited effectiveness in specific scenarios, indicating the need to explore more advanced approaches such as t-SNE or UMAP. While models such as SVM and ANN demonstrated high predictive accuracy, their limited interpretability remains a challenge; therefore, integrating explainable artificial intelligence tools such as LIME or SHAP is essential to enhance clinical transparency and trust in AI-assisted diagnostic systems. Finally, the absence of an external validation dataset represents a further limitation, as external validation is crucial for confirming model performance in independent populations. 

## 5. Conclusions

This study demonstrates that machine learning algorithms can serve as powerful diagnostic support tools in the classification of osteoporosis and osteopenia. In particular, data-driven feature selection methods such as ANOVA, CHI2, and MRMR provided higher classification accuracies than traditional diagnostic regions, especially when combined with algorithms such as SVM and k-NN, which achieved high overall performance. Although commonly used BMD regions in clinical practice also yielded strong classification results, statistically selected features exhibited superior discriminative power.

In this context, integrating machine learning models supported by data-based feature selection into clinical decision support systems may contribute significantly to early diagnosis and treatment planning, ultimately reducing fracture risk and alleviating the burden on healthcare systems. Future studies are recommended to develop multivariate models enriched with larger and more diverse datasets, incorporating genetic, lifestyle, and hormonal variables to further enhance model accuracy and clinical applicability.

## Figures and Tables

**Table 1:** Demographic and Clinical Characteristics of the Study Population.

Characteristic	Healthy (*n* = 40)	Osteopenia (*n* = 52)	Osteoporosis (*n* = 50)	*p*-Value
Age (years)	55.2 ± 8.3	61.7 ± 7.9	64.5 ± 9.1	<0.001
Height (cm)	162.4 ± 6.2	158.1 ± 5.8	155.3 ± 6.5	0.003
Weight (kg)	68.5 ± 10.1	65.2 ± 9.7	60.8 ± 8.4	0.012
BMI (kg/m^2^)	26.0 ± 3.1	25.8 ± 3.4	24.1 ± 2.9	0.078
Lumbar Spine BMD (L1–L4) (g/cm^2^)	1.12 ± 0.15	0.92 ± 0.11	0.72 ± 0.09	<0.001
Femoral Neck BMD (g/cm^2^)	0.95 ± 0.12	0.78 ± 0.10	0.61 ± 0.08	<0.001

**Table 2:** Classifier Performance Results Obtained Using All Features:.

		Predicted Class					Accuracy
DT [All Features]		Normal	Osteopenia	Osteoporosis	TPR (%)	FNR (%)	
True class	Normal	37	5	0	88.10%	11.90%	82.90%
	Osteopenia	5	42	3	84.00%	16.00%	
	Osteoporosis	1	10	37	77.10%	22.90%	
LDA [all features]		Normal	Osteopenia	Osteoporosis	TPR (%)	FNR (%)	
True class	Normal	16	26	0	38.10%	61.90%	70.00%
	Osteopenia	4	45	1	90.00%	10.00%	
	Osteoporosis	0	11	37	77.10%	22.90%	
NB [all features]		Normal	Osteopenia	Osteoporosis	TPR (%)	FNR (%)	
True class	Normal	39	3	0	92.90%	7.10%	82.90%
	Osteopenia	7	37	6	74.00%	26.00%	
	Osteoporosis	0	8	40	83.30%	16.70%	
SVM [all features]		Normal	Osteopenia	Osteoporosis	TPR (%)	FNR (%)	
True class	Normal	40	2	0	95.20%	4.80%	75.00%
	Osteopenia	24	24	2	48.00%	52.00%	
	Osteoporosis	3	4	41	85.40%	14.60%	
k-NN [all features]		Normal	Osteopenia	Osteoporosis	TPR (%)	FNR (%)	
True class	Normal	17	25	0	40.50%	59.50%	72.90%
	Osteopenia	6	43	1	86.00%	14.00%	
	Osteoporosis	1	5	42	87.50%	12.50%	
ANN [all features]		Normal	Osteopenia	Osteoporosis	TPR (%)	FNR (%)	
True class	Normal	18	24	0	42.90%	57.10%	74.30%
	Osteopenia	4	44	2	88.00%	12.00%	
	Osteoporosis	0	6	42	87.50%	12.50%	

**Table 3:** Classification Performances Obtained Using L1–L4, Femoral Neck and Total Femur Features.

		Predicted Class					Accuracy
DT [3 Features]		Normal	Osteopenia	Osteoporosis	TPR (%)	FNR (%)	
True class	Normal	40	2	0	92.20%	4.80%	87.90%
	Osteopenia	5	42	3	84.00%	16.00%	
	Osteoporosis	3	4	41	85.40%	14.60%	
LDA [3 features]		Normal	Osteopenia	Osteoporosis	TPR (%)	FNR (%)	
True class	Normal	34	8	0	81.00%	19.00%	87.10%
	Osteopenia	5	42	3	84.00%	16.00%	
	Osteoporosis	1	1	46	95.80%	4.20%	
NB [3 features]		Normal	Osteopenia	Osteoporosis	TPR (%)	FNR (%)	
True class	Normal	40	2	0	95.20%	4.80%	89.30%
	Osteopenia	7	39	4	78.00%	22.00%	
	Osteoporosis	1	1	46	95.80%	4.20%	
SVM [3 features]		Normal	Osteopenia	Osteoporosis	TPR (%)	FNR (%)	
True class	Normal	38	4	0	90.50%	9.50%	90.70%
	Osteopenia	5	43	2	86.00%	14.00%	
	Osteoporosis	1	1	46	95.80%	4.20%	
k-NN [3 features]		Normal	Osteopenia	Osteoporosis	TPR (%)	FNR (%)	
True class	Normal	41	1	0	97.60%	2.40%	90.00%
	Osteopenia	6	42	2	84.00%	16.00%	
	Osteoporosis	1	4	43	89.60%	10.40%	
ANN [3 features]		Normal	Osteopenia	Osteoporosis	TPR (%)	FNR (%)	
True class	Normal	36	4	2	85.70%	14.30%	85.00%
	Osteopenia	5	39	6	78.00%	22.00%	
	Osteoporosis	0	4	44	91.70%	8.30%	

**Table 4:** Classification Performances Obtained Using L3–L4, Femoral Neck and Femoral Wards Features.

		Predicted Class					Accuracy
DT [3 Features]		Normal	Osteopenia	Osteoporosis	TPR (%)	FNR (%)	
True class	Normal	39	3	0	92.90%	7.10%	82.90%
	Osteopenia	7	38	5	76.00%	24.00%	
	Osteoporosis	0	9	39	81.20%	18.80%	
LDA [3 features]		Normal	Osteopenia	Osteoporosis	TPR (%)	FNR (%)	
True class	Normal	36	6	0	85.70%	14.30%	78.60%
	Osteopenia	5	34	11	68.00%	32.00%	
	Osteoporosis	0	8	40	83.30%	16.70%	
NB [3 features]		Normal	Osteopenia	Osteoporosis	TPR (%)	FNR (%)	
True class	Normal	38	4	0	90.50%	9.50%	80.00%
	Osteopenia	8	35	7	70.00%	30.00%	
	Osteoporosis	0	9	39	81.20%	18.80%	
SVM [3 features]		Normal	Osteopenia	Osteoporosis	TPR (%)	FNR (%)	
True class	Normal	36	6	0	85.70%	14.30%	8.36%
	Osteopenia	5	38	7	76.00%	24.00%	
	Osteoporosis	0	5	43	89.60%	10.40%	
k-NN [3 features]		Normal	Osteopenia	Osteoporosis	TPR (%)	FNR (%)	
True class	Normal	36	6	0	85.70%	14.30%	77.90%
	Osteopenia	8	36	6	72.00%	28.00%	
	Osteoporosis	0	11	37	77.10%	22.90%	
ANN [3 features]		Normal	Osteopenia	Osteoporosis	TPR (%)	FNR (%)	
True class	Normal	38	4	0	90.50%	9.50%	75.70%
	Osteopenia	9	29	12	58.00%	42.00%	
	Osteoporosis	0	9	39	81.20%	18.80%	

**Table 5:** Classification Performance Based on CHI2 Feature Selection: Accuracy, TPR, and FNR Values.

		Predicted Class					Accuracy
DT [3 Features]		Normal	Osteopenia	Osteoporosis	TPR (%)	FNR (%)	
True class	Normal	41	1	0	97.60%	2.40%	90.00%
	Osteopenia	3	43	4	86.00%	14.00%	
	Osteoporosis	0	6	42	87.50%	12.50%	
LDA [3 features]		Normal	Osteopenia	Osteoporosis	TPR (%)	FNR (%)	
True class	Normal	33	8	1	78.60%	21.40%	83.60%
	Osteopenia	7	39	4	78.00%	22.00%	
	Osteoporosis	0	3	45	93.80%	6.20%	
NB [3 features]		Normal	Osteopenia	Osteoporosis	TPR (%)	FNR (%)	
True class	Normal	41	1	0	97.60%	2.40%	89.30%
	Osteopenia	5	41	4	82.00%	18.00%	
	Osteoporosis	0	5	43	89.60%	10.40%	
SVM [3 features]		Normal	Osteopenia	Osteoporosis	TPR (%)	FNR (%)	
True class	Normal	42	0	0	100.00%	0.00%	91.40%
	Osteopenia	5	42	3	84.00%	16.00%	
	Osteoporosis	0	4	44	91.70%	8.30%	
k-NN [3 features]		Normal	Osteopenia	Osteoporosis	TPR (%)	FNR (%)	
True class	Normal	42	0	0	100.00%	0.00%	90.70%
	Osteopenia	4	41	5	82.00%	18.00%	
	Osteoporosis	1	3	44	91.70%	8.30%	
ANN [3 features]		Normal	Osteopenia	Osteoporosis	TPR (%)	FNR (%)	
True class	Normal	41	1	0	97.60%	2.40%	90.00%
	Osteopenia	4	41	5	82.00%	18.00%	
	Osteoporosis	1	3	44	91.70%	8.30%	

**Table 6:** Classification Performance Based on ANOVA Feature Selection: Accuracy, TPR, and FNR Values.

		Predicted Class					Accuracy
DT [3 Features]		Normal	Osteopenia	Osteoporosis	TPR (%)	FNR (%)	
True class	Normal	41	1	0	97.60%	2.40%	91.40%
	Osteopenia	3	43	4	86.00%	14.00%	
	Osteoporosis	0	4	44	91.70%	8.30%	
LDA [3 features]		Normal	Osteopenia	Osteoporosis	TPR (%)	FNR (%)	
True class	Normal	35	7	0	83.30%	16.70%	85.00%
	Osteopenia	6	39	5	78.00%	22.00%	
	Osteoporosis	0	3	45	93.80%	6.20%	
NB [3 features]		Normal	Osteopenia	Osteoporosis	TPR (%)	FNR (%)	
True class	Normal	41	1	0	97.60%	2.40%	90.70%
	Osteopenia	4	43	3	86.00%	14.00%	
	Osteoporosis	0	5	43	89.60%	10.40%	
SVM [3 features]		Normal	Osteopenia	Osteoporosis	TPR (%)	FNR (%)	
True class	Normal	42	0	0	100.00%	0.00%	94.30%
	Osteopenia	4	45	1	90.00%	10.00%	
	Osteoporosis	0	3	45	93.80%	6.20%	
k-NN [3 features]		Normal	Osteopenia	Osteoporosis	TPR (%)	FNR (%)	
True class	Normal	41	1	0	97.60%	2.40%	92.10%
	Osteopenia	4	43	3	86.00%	14.00%	
	Osteoporosis	0	3	45	93.80%	6.20%	
ANN [3 features]		Normal	Osteopenia	Osteoporosis	TPR (%)	FNR (%)	
True class	Normal	41	1	0	97.60%	2.40%	91.40%
	Osteopenia	4	43	3	86.00%	14.00%	
	Osteoporosis		4	44	91.70%	8.30%	

**Table 7:** Classification Performance Based on Kruskal–Wallis Feature Selection: Accuracy, TPR, and FNR Values.

		Predicted Class					Accuracy
DT [3 Features]		Normal	Osteopenia	Osteoporosis	TPR (%)	FNR (%)	
True class	Normal	39	3	0	92.90%	7.10%	84.30%
	Osteopenia	4	43	3	86.00%	14.00%	
	Osteoporosis	0	12	36	75.00%	25.00%	
LDA [3 features]		Normal	Osteopenia	Osteoporosis	TPR (%)	FNR (%)	
True class	Normal	32	10	0	76.20%	23.80%	79.30%
	Osteopenia	3	39	8	78.00%	22.00%	
	Osteoporosis	0	8	40	83.30%	16.70%	
NB [3 features]		Normal	Osteopenia	Osteoporosis	TPR (%)	FNR (%)	
True class	Normal	39	3	0	92.90%	7.10%	83.60%
	Osteopenia	4	40	6	80.00%	20.00%	
	Osteoporosis	0	10	38	79.20%	20.80%	
SVM [3 features]		Normal	Osteopenia	Osteoporosis	TPR (%)	FNR (%)	
True class	Normal	39	3	0	92.90%	7.10%	85.00%
	Osteopenia	4	40	6	80.00%	20.00%	
	Osteoporosis	0	8	40	83.30%	16.70%	
k-NN [3 features]		Normal	Osteopenia	Osteoporosis	TPR (%)	FNR (%)	
True class	Normal	40	2	0	95.20%	4.80%	85.00%
	Osteopenia	2	42	6	84.00%	16.00%	
	Osteoporosis		11	37	77.10%	22.90%	
ANN [3 features]		Normal	Osteopenia	Osteoporosis	TPR (%)	FNR (%)	
True class	Normal	39	3	0	92.90%	7.10%	78.60%
	Osteopenia	8	35	7	70.00%	30.00%	
	Osteoporosis	0	12	36	75.00%	25.00%	

**Table 8:** Accuracy Comparison of Classifiers Based on Different Feature Selection Methods.

Feature Space						
All features	DT	LDA	NB	SVM	k-NN	ANN
	82.90%	70.00%	82.90%	75.00%	72.90%	74.30%
Standard Evaluation	87.90%	87.10%	89.30%	90.70%	90.00%	85.00%
MRMR	82.90%	78.60%	80.00%	83.60%	85.00%	75.70%
CHI2	90.00%	83.60%	89.30%	91.40%	90.70%	90.00%
ANOVA	91.40%	85.00%	90.70%	94.30%	92.10%	91.40%
Kruskal–Wallis	84.30%	79.30%	83.60%	85.00%	85.00%	78.60%

**Table 9:** Classification metrics for SVM + ANOVA-selected features.

Class	Precision	Recall	F1-Score
Normal	0.913	1.00	0.954
Osteopenia	0.938	0.90	0.918
Osteoporosis	0.978	0.938	0.957
Macro Avg	0.943	0.946	0.943

## Data Availability

The data are available from the corresponding author upon reasonable request. Access to the dataset is restricted due to hospital privacy regulations.

## References

[1] Yoo J.-H., Moon S.-H., Ha Y.-C., Lee D.Y., Gong H.S., Palrk S.Y., Yang K.H. (2015). Osteoporotic fracture: 2015 position statement of the Korean society for bone and mineral research. J. Bone Metab..

[2] Svedbom A., Hernlund E., Ivergård M., Compston J., Cooper C., Stenmark J., McCloskey E.V., Jönsson B., Kanis J.A., the EU review panel of the IOF (2013). Osteoporosis in the European Union: A compendium of country-specific reports. Arch. Osteoporos..

[3] Kanis J., Cooper C., Rizzoli R., Abrahamsen B., Al-Daghri N.M., Brandi M.L., Cannata-Andia J., Cortet B., Dimai H.P., Ferrari S. (2017). Identification and management of patients at increased risk of osteoporotic fracture: Outcomes of an ESCEO expert consensus meeting. Osteoporos. Int..

[4] Tangen K.M., Leval R., Mehta A.I., Linninger A.A. (2017). Computational and in vitro experimental investigation of intrathecal drug distribution: Parametric study of the effect of injection volume, cerebrospinal fluid pulsatility, and drug uptake. Anesth. Analg..

[5] Kanis J.A., Kanis J. (1994). Assessment of fracture risk and its application to screening for postmenopausal osteoporosis: Synopsis of a WHO report. Osteoporos. Int..

[6] Erjiang E., Yang L., Dempsey M., Brennan A., Yu M., Chan W.P., Whelan B., Silke C., O′SUllivan M., Rooney B. (2021). Machine learning can improve clinical detection of low BMD: The DXA-HIP study. J. Clin. Densitom..

[7] Kang J.-W., Park C., Lee D.-E., Yoo J.-H., Kim M. (2023). Prediction of bone mineral density in CT using deep learning with explainability. Front. Physiol..

[8] Roski F., Hammel J., Mei K., Baum T., Kirschke J.S., Laugerette A., Kopp F.K., Bodden J., Pfeiffer D., Pfeiffer F. (2019). Bone mineral density measurements derived from dual-layer spectral CT enable opportunistic screening for osteoporosis. Eur. Radiol..

[9] Nayak S., Roberts M.S., Greenspan S.L. (2011). Cost-effectiveness of different screening strategies for osteoporosis in postmenopausal women. Ann. Intern. Med..

[10] Liu Y., Meng X.-H., Wu C., Su K.-J., Liu A., Tian Q., Zhao L.-J., Qiu C., Luo Z., I Gonzalez-Ramirez M. (2024). Variability in performance of genetic-enhanced DXA-BMD prediction models across diverse ethnic and geographic populations: A risk prediction study. PLoS Med..

[11] Miura K., Tanaka S.M., Chotipanich C., Chobpenthai T., Jantarato A., Khantachawana A. (2024). Osteoporosis prediction using machine-learned optical bone densitometry data. Ann. Biomed. Eng..

[12] Fodeh S., Wang R., Murphy T.E., Kidwai-Khan F., Leo-Summers L.S., Tessier-Sherman B., Hsieh E., Womack J.A. (2024). BoneScore: A natural language processing algorithm to extract bone mineral density data from DXA scans. Health Inform. J..

[13] Bezerra G.M., Ohata E.F., Loureiro L.L., Bittencourt V.Z., Capistrano V.L.M., da Rocha A.R., Filho P.P.R. (2024). Estimation of Bone Mineral Density using Machine Learning and SHapley Additive exPlanations. Proceedings of the 2024 IEEE 37th International Symposium on Computer-Based Medical Systems (CBMS).

[14] Li J., Zhang P., Xu J., Zhang R., Ren C., Yang F., Li Q., Dong Y., Huang C., Zhao J. (2025). Prediction of bone mineral density based on computer tomography images using deep learning model. Gerontology.

[15] Cosman F., de Beur S.J., LeBoff M.S., Lewiecki E.M., Tanner B., Randall S., Lindsay R. (2014). Clinician’s guide to prevention and treatment of osteoporosis. Osteoporos. Int..

[16] Sinha R., Bukhari M. (2014). OP0292 The Diagnosis of osteoporosis using BMD and T score measurements at specific skeletal sites. Ann. Rheum. Dis..

[17] Woodson G. (2000). Dual X-ray absorptiometry T-score concordance and discordance between hip and spine measurement sites. J. Clin. Densitom..

[18] Chen P., Miller P.D., Binkley N.C., Kendler D.L., Wong M., Krohn K. (2008). Use of lowest single lumbar spine vertebra bone mineral density T-score and other T-score approaches for diagnosing osteoporosis and relationships with vertebral fracture status. J. Clin. Densitom..

[19] Özdemir O., Yasrebi S., Kutsal Y. (2015). Evaluation of Concordance between Hip and Spine T Scores in the Diagnosis of Osteoporosis in Men Over Age of Fifty. Turk. Osteoporoz. Derg. Turk. J. Osteoporos..

[20] Vendrami C., Shevroja E., Gatineau G., Rodriguez E.G., Olivier L., Hans D. (2023). Comparison between Horizon A System and Lunar iDXA in Bone Assessment and Osteoporosis Diagnosis: The OsteoLaus Cohort. J. Clin. Densitom..

[21] Padlina I., Gonzalez-Rodriguez E., Hans D., Metzger M., Stoll D., Aubry-Rozier B., Lamy O. (2017). The lumbar spine age-related degenerative disease influences the BMD not the TBS: The Osteolaus cohort. Osteoporos. Int..

[22] Bergil E., Bozkurt M.R., Oral C. (2021). An evaluation of the channel effect on detecting the preictal stage in patients with epilepsy. Clin. EEG Neurosci..

[23] Bergil E. (2023). Application of Dimension Reduction Methods for Stress Detection. Int. J. Pioneer. Technol. Eng..

[24] Kumar A., Kaur A., Singh P., Driss M., Boulila W. (2023). Efficient Multiclass Classification Using Feature Selection in High-Dimensional Datasets. Electronics.

[25] Wang Z., Chen H., Yang X., Wan J., Li T., Luo C. (2023). Fuzzy rough dimensionality reduction: A feature set partition-based approach. Inf. Sci..

[26] Miranda D., Olivares R., Munoz R., Minonzio J.-G. (2022). Improvement of patient classification using feature selection applied to bidirectional axial transmission. IEEE Trans. Ultrason. Ferroelectr. Freq. Control.

[27] Khanna V.V., Chadaga K., Sampathila N., Chadaga R., Prabhu S., Swathi S.K., Jagdale A.S., Bhat D. (2023). A decision support system for osteoporosis risk prediction using machine learning and explainable artificial intelligence. Heliyon.

[28] Calitis M. (2024). Risk Factor Identification in Osteoporosis Using Unsupervised Machine Learning Techniques. arXiv.

[29] Cha Y., Seo S.H., Kim J.-T., Kim J.-W., Lee S.-Y., Yoo J.-I. (2023). Osteoporosis feature selection and risk prediction model by machine learning using a cross-sectional database. J. Bone Metab..

[30] Oka R., Ohira M., Suzuki S., Yoshida T., Koide H., Tanaka T., Tatsuno I. (2018). Fracture risk assessment tool (FRAX) and for the diagnosis of osteoporosis in Japanese middle-aged and elderly women: Chiba bone survey. Endocr. J..

[31] Sheng Y.-H., Wu T.-Y., Liaw C.-K., Hsiao S.-H., Kuo K.-L., Tsai C.-Y. (2024). Real world fracture prediction of fracture risk assessment tool (FRAX), osteoporosis self-assessment tool for Asians (OSTA) and one-minute osteoporosis risk test: An 11-year longitudinal study. Bone Rep..

[32] Lehmann O., Mineeva O., Veshchezerova D., Häuselmann H., Guyer L., Reichenbach S., Lehmann T., Demler O., Everts-Graber J., The Swiss Osteoporosis Registry Study Group (2024). Fracture risk prediction in postmenopausal women with traditional and machine learning models in a nationwide, prospective cohort study in Switzerland with validation in the UK Biobank. J. Bone Miner. Res..

